# Influence of time factor and albuminuria on characteristics of patients with type 2 diabetes Mellitus before, during and 1 year after COVID-19 recovery

**DOI:** 10.1186/s13098-023-01104-y

**Published:** 2023-06-13

**Authors:** Mohammed Ali Gameil, Rehab Elsayed Marzouk, Ahmed Hassan El-Sebaie, Ahmed Ahmed Ahmed Eldeeb

**Affiliations:** 1 Endocrinology Unit, Internal Medicine Department, Faculty of Medicine, Mansoura University, Mansoura, Dakahlia Egypt; 2 Lecturer of Medical Biochemistry, Medical Biochemistry Department, Faculty of Medicine, Helwan University, Helwan, 0000-0002, 5551- 1540 Cairo Egypt; 3 Clinical Pathology Department, Faculty of Medicine, Mansoura University, Mansoura, Dakahlia Egypt; 4 Associate professor of Internal medicine, Nephrology Unit, Internal Medicine Department, Faculty of Medicine, Mansoura University, Mansoura, 0000-0002, 3238-3064 Dakahlia Egypt

**Keywords:** COVID-19 recovery, Type 2 diabetes Mellitus, Albuminuria, Time factor, One year

## Abstract

**Background:**

The potential effects of time factor and albuminuria on the morbid alterations in patients with type 2 diabetes (T2D) and COVID-19 are still unclear. We aimed to address the morbid alterations and the potential effects of time factor and albuminuria on the patients’ characteristics before, during, and 1 year after COVID-19 recovery.

**Methods:**

83 patients with T2D were included, at Mansoura University Hospital, Egypt (July 2021-December 2021). Data of detailed history, physical examination, laboratory tests were recruited from files of the patients. Diagnosis and resolution of COVID-19 were established by Real time polymerase chain reaction (RT-PCR) test of SARS-CoV2. Complete blood count (CBC), renal and hepatic function tests, multiple measures of morning spot urine albumin to creatinine ratio (urine ACR), glycosylated hemoglobin (HBA1c), lipid profile, erythrocyte sedimentation rate (ESR), C-reactive protein (CRP), Ferritin, neutrophil to lymphocyte ratio (NLR), vitamin D3, intact parathyroid hormone (intact PTH), serum calcium were applied to all participants.

**Results:**

Our participants had a mean age of 45 years, 60.2% male, 56.6% were hospitalized, and 25.3% were admitted to ICU for severe COVID-19. Albuminuria was prevalent in 71.1% before, 98.8% during, and 92.8% after COVID-19 recovery. Patients with albuminuria showed older age, longer duration of T2D, more frequent severe COVID-19 and hospitalization (p = 0.03, p < 0.001, p = 0.023& p = 0.025) respectively. Body mass index (BMI), mean arterial blood pressure, ESR, CRP, ferritin, NLR, HBA1c, triglycerides to high-density lipoprotein cholesterol (TG/HDL-C) ratio, vitamin D3, serum calcium, alkaline phosphatase (ALP), hepatic aminotransferases, and urine ACR showed significant alterations throughout the study (p < 0.001 for all). Although the interaction between time and albuminuria showed non-significant effect on all studied parameters, we noticed relevant main effects of time factor on Body mass index (BMI), HBA1c, glomerular filtration rate (eGFR), TG/HDL ratio, NLR, vitamin D3, (p < 0.001 for all). Moreover, albuminuria showed main effects on BMI, serum creatinine, and intact PTH (p = 0.019, 0.005 & <0.001), respectively.

**Conclusion:**

The characteristics of patients with T2D significantly altered throughout the study. Time factor and albuminuria exerted relevant main effects on the patients’ characteristics without significant effect of their interaction.

## Background

Human beings have been afflicted by a catastrophic pandemic of coronavirus disease (COVID-19). COVID-19 has enigmatic clinical features and complications from mild respiratory tract infection to critical illness with multiple organ failure [1]. SARS-CoV2 is a novel coronavirus with a structural similarity to severe acute respiratory syndrome coronavirus (SARS-CoV); causative virus of 2003 epidemic [2]. Angiotensin-converting enzyme 2 (ACE2) is the cellular receptor for SARS-CoV and SARS-CoV2. ACE2 is enormously dispensed at the lungs, kidneys, small intestine, heart, genitals, pancreas, liver, blood cells, vascular endothelium, thyroid, and adrenal glands [3]. The interplay between diabetes mellitus (DM) and COVID-19 conveys serious hazards. Obviously, Diabetes mellitus represents a leading cause of deleterious outcomes of COVID-19. Diabetes mellitus associated hyperglycemia, pro-inflammatory and hypercoagulability state, microvascular and macrovascular complications, and co-morbidities like obesity, hypertension, dyslipidemia, and atherosclerotic cardiovascular diseases are major risk factors for poor outcomes of COVID-19 [4, 5]. HBA1c independently determined the risk of mortality from COVID-19 with a positive linear relationship. The vulnerability to infections increased with DM with devastating consequences. Constellation of hyperglycemia, COVID-19-related cytokine storm, overwhelming stress, inflammatory species, and impaired immune response may induce poor outcomes [6–10]. The abundant expression of ACE2 in the apical membrane of the epithelial cells at the proximal renal tubules enables SARS-CoV2 to tackle the kidneys with perilous effects like proteinuria, hematuria, and acute kidney injury [11, 12]. Co-existing diabetic kidney disease and COVID-19 may induce devastating consequences of acute kidney injury and mortality risk [13, 14]. On the other side, SARS-CoV2 and therapeutic agents used for COVID-19 may disrupt glycemic control, and adversely affect pancreatic beta cell functions and insulin action [15].

In literature, multiple studies were conducted to detect COVID-19 related morbid alterations during and following COVID-19 in patients with and without DM. In the current study, we aimed to track and address clinical and biochemical alterations in patients with type 2 diabetes mellitus (T2D) before, during, and 1 year after COVID-19 recovery. Our secondary objective was to detect the potential effects of time factor, albuminuria, and their interaction on the characteristics of patients with T2D throughout the aforementioned time points.

## Methods

An observational longitudinal retrospective study was conducted at the outpatient department of Mansoura University Hospital during the period from July 2021 to December 2021. Following official ethical approval and signing a written consent to participate, 139 patients with T2D and prior history of COVID-19 were recruited. Owing to missing data of some studied parameters before or during COVID-19, 83 patients owned complete data of predefined parameters. Detailed clinical history of duration of DM, smoking status, pregnancy status, associated comorbidities such as systemic hypertension, dyslipidemia, non-alcoholic fatty liver disease (NAFLD), coronary artery disease (CAD), peripheral arterial disease, and therapeutic history (e.g.; use of rennin angiotensin aldosterone inhibitors; RAAS-I). Physical examination and anthropometric measures were recorded. Sever COVID-19 was identified by the need for urgent hospital admission, intensive care unit (ICU) admission for desaturation and urgent oxygen therapy. Laboratory investigations included complete blood count (CBC), serum creatinine, blood urea nitrogen (BUN), estimated glomerular filtration rate (eGFR), multiple measures of morning spot urine albumin to creatinine ratio (urine ACR), alanine aminotransferase (ALT), aspartate aminotransferase (AST), gamma-glutamyl trans-peptidase (GGT), serum bilirubin, albumin, alkaline phosphatase (ALP), glycosylated hemoglobin (HBA1c), serum uric acid, fasting lipid profile, erythrocyte sedimentation rate (ESR), C-reactive protein (CRP), Ferritin, neutrophil to lymphocyte ratio (NLR), derived neutrophil to lymphocyte ratio (dNLR), vitamin D3, intact parathyroid hormone (intact PTH), serum calcium, phosphorus, and magnesium. Albuminuria was confirmed by at least 2 samples of morning spot urine albumin to creatinine ratio collected in 2 different occasions over 3 months as documented in patients’ files before and after COVID-19. During COVID-19, albuminuria was recorded and documented in the files of the patients. Participants were subdivided into 2 groups; with and without albuminuria according to the cutoff value of the American Diabetes Association whereas albuminuria if urine ACR more than 30 mg/g [16]. Real time polymerase chain reaction (RT-PCR) test of SARS-CoV2 was used to confirm diagnosis as well as resolution of COVID-19. An overnight fasting venous blood samples were withdrawn from participants. Blood samples were collected, centrifuged and the sera were used for estimation of creatinine and urea levels, lipid profile. Commercial kits supplied by (BioSystems, Egypt) and (Human® company, Egypt) were utilized for serum creatinine, urea, microalbuminuria, and plasma lipid estimation. Blood samples were collected in EDTA tube for determination glycosylated hemoglobin (HbA1c), vitamin D3, and intact PTH by (electrochemiluminescence analyzer cobas e411). Automated chemistry analyzer Beckman was used for assay of serum calcium, magnesium, and phosphorus. The Modification of Diet in Renal Disease (MDRD) formula was used for the eGFR estimation [17]. Hematology analyzer Rubby Abbott, Latex Biomed, and Westergren tube method were used for CBC, CRP, and ESR assay, respectively. Real time polymerase chain reaction for SARS-CoV2 was conducted with (PCR Thermo Fisher Scientific Inc., Waltham, MA, USA). Patients with endocrine disorders, autoimmune diseases, rheumatologic, neoplastic, hematologic diseases, and chronic inflammatory diseases were excluded. Patients with pregnancy, decompensated liver, renal, pulmonary or cardiac functions were ruled-out. Patients with a history of acute infections coinciding registered data before or following COVID-19 were excluded. Other causes of transient albuminuria before and after COVID-19 like pyrexia, heavy exercise, high protein intake, menstruation, urinary tract infection, and non-steroidal anti-inflammatory drug users were ruled-out.

### Ethical approval

The official approval was obtained from the Institutional Review Board (IRB) for the Clinical Research Committee of Mansoura University with approval number ***(No.R.20.06.1158) on 15/9/2020***. All procedures performed were in accordance with the ethical standards of the institutional research committee and with the 1964 Helsinki Declaration and later versions. Written consent for participation was approved by the IRB and obtained from all participants before enrolment.

### Statistical analysis

Data were entered and analyzed using the IBM-SPSS software (IBM Corp. Released 2017. IBM SPSS Statistics for Windows, Version 25.0. Armonk, NY: IBM Corp.). Qualitative data were expressed as frequency (percentage) and compared by the chi-square test. Quantitative data were expressed as mean ± standard deviation (SD) due to its normality (Shapiro-Wilk’s test, p > 0.050) and no significant outliers. Quantitative data were compared between the three time points; before, during and after COVID-19 by one-way repeated measure ANOVA test. Cochran’s Q test was applied in analysis of categorical repeatedly measured parameter. Independent-Samples t-test, Chi-Square test and Fisher’s exact test were used for analysis of demographic data of albuminuria and non-albuminuria groups. The two-way mixed ANOVA was run to determine the effects of the interaction between albuminuria and time factor on studied parameters. Variables without significant effect of the interaction between time factor and albuminuria were subjected to the two-way mixed ANOVA to evaluate the main effects of time factor regardless of albuminuria and the main effects of albuminuria regardless of the time point. Significant values were considered at p ≤ 0.05 level.

## Results

Table (1) shows the demographic differences between participants with and without albuminuria. Patients with baseline albuminuria exhibited significantly older age (p = 0.03), longer duration of T2D (p < 0.001), hypertension with RAAS-I use (p = 0.017), and more frequent severe COVID-19 that necessitated hospitalization and ICU admission (p = 0.023 and 0.025), respectively versus participants without albuminuria.

**Table 1:** Demographic characteristics of patients with and without albuminuria

Characteristic	Without albuminuria	With albuminuria	P-value
	N = 24	N = 59	
**Age** (years)	43.5 ± 4.7	46.5 ± 6.1	0.030* (t)
**Gender**			0.471 (χ)
**Male** **Female**	13 (52.2%) 11 (45.8%)	37 (62.7%) 22 (37.3%)	
**Current smoking**	3 (12.5%)	18 (30.5%)	0.087 (χ)
**Hypertension (RAAS-I use)**	9 (37.5%)	39 (66.1%)	**0.017** (χ)
**Dyslipidaemia**	21 (87.5%)	52 (88.1%)	1.000 (F)
**DM duration** (years)	4.3 ± 1.3	5.8 ± 2.2	< 0.001* (t)
**Severe COVID-19** (ICU)	2 (8.3%)	19 (32.3%)	0.023* (χ)
**Hospitalization** (O2 sat < 92%)	9 (37.5%)	38 (64.4%)	0.025* (χ)

¶ Test of significance: (t): Independent-Samples t-test. (χ): Chi-Square test. (F): Fisher’s exact test. †Data expression: Mean ± SD for age and DM duration, and N (%) for qualitative data. * Significant value: p ≤ 0.05

One-way repeated measure ANOVA test revealed statistically significant differences in clinical and biochemical characteristics of participants before, during and after COVID-19. Significant alterations were relevant for Bodyweight, body mass index (BMI), mean arterial blood pressure (mean ABP), ESR, CRP, ferritin, NLR, dNLR, absolute neutrophil count (ANC), lymphocytic count, vitamin D3, intact PTH, HBA1c, TG, TG/HDL-C ratio, serum calcium, magnesium, phosphorus, ALP, ALT, AST, GGT, BUN, eGFR, serum creatinine and urine albumin/creatinine ratio (p < 0.001 for all). (See Table 2). Table (3) shows the categorical repeatedly measured parameters before, during and after COVID-19. Albuminuria was prevalent in 71.1% of patients before COVID-19, exacerbated to 98.8% during COVID-19, and finally regress to 92.8% after COVID-19 (p < 0.001). TG/HDL-C ratio ≥ 3 was statistically and significantly lowered after COVID-19 than before or during COVID-19 (p = 0.002). Conversely, the prevalence of use of RAAS-I statistically and significantly increased after COVID-19 recovery than before or during COVID-19 (p < 0.001). In Table (4), two-way mixed ANOVA test was run to determine the effect of the interaction between albuminuria (with and without albuminuria) and time (before, during, and after COVID-19) on the studied parameters. All studied variables showed non-significant effect of the interaction between albuminuria and time factor. Table (5) shows the main effects of albuminuria regardless of time factor and the main effects of time factor regardless of albuminuria on studied parameters. The main effects of albuminuria were significantly relevant for BMI, BUN, serum creatinine, intact PTH, ALP, and calcium; (p = 0.019, 0.004, 0.005, < 0.001, 0.004, & 0.037), respectively. Meanwhile, the main effects of time were relevant for BMI, HBA1c, BUN, serum creatinine, eGFR, serum uric acid, TG, TG/HDL ratio, VLDL-cholesterol, NLR, dNLR, ANC, vitamin D3, ALP, calcium, magnesium, and phosphorus (p < 0.001 for all). Furthermore, mean ABP, HDL- cholesterol and LDL-cholesterol were significantly affected by time factor (p = 0.003, 0.015 and 0.025), respectively.

**Table 2:** Clinical and laboratory parameters before, during, and 1 year after COVID-19 recovery

Parameter	Before	During	After	F-value	P-value	Partial η^2^
**Body weight** (kg)	86.4 ± 8.3 a	87.9 ± 8.7 b	89.5 ± 8.5 c	148.932	< 0.001*	0.645
**BMI** (kg/m^2^)	29.4 ± 3.4 a	29.9 ± 3.6 b	30.3 ± 3.4 c	63.441	< 0.001*	0.436
**SBP** (mmHg)	134.9 ± 8.3 a	131.9 ± 9 b	134 ± 9.3 a, b	5.109	0.009*	0.059
**DBP** (mmHg)	84.6 ± 5.8 a	81.4 ± 5.5 b	84.8 ± 7.6 a	9.363	< 0.001*	0.102
**MAP** (mmHg)	101.4 ± 6.1 a	98.2 ± 6.1 b	101.2 ± 7.6 a	9.458	< 0.001*	0.103
**ESR** (mm/h)	12.6 ± 3.9 a	58.2 ± 15.7 b	25.4 ± 7.8 c	513.902	< 0.001*	0.862
**CRP** (mg/L)	7.6 ± 1.8 a	61.6 ± 28.8 b	16.2 ± 6.9 c	259.125	< 0.001*	0.760
**Ferritin** (ng/mL)	51.8 ± 24.2 a	220.4 ± 76.2 b	113.2 ± 25.7 c	369.308	< 0.001*	0.818
**WBCs count** (× 10^9^/L)	6.36 ± 1.6 a	7.57 ± 3.3 b	6.5 ± 1.9 a	11.706	< 0.001*	0.125
**ANC** (× 10^3^/mcL)	3.89 ± 1.1 a	6.15 ± 3.3 b	3.6 ± 1.2 a	51.151	< 0.001*	0.384
**Polymorphs %**	61.1 ± 6.4 a	78.6 ± 12.9 b	55.5 ± 9.6 c	149.593	< 0.001*	0.646
**Lymphocyte %**	30.1 ± 6.7 a	14.5 ± 11.4 b	33.3 ± 7.8 c	140.059	< 0.001*	0.631
**NLR**	2.18 ± 0.69 a	10.25 ± 7.8 b	1.85 ± 0.85 c	91.906	< 0.001*	0.528
**dNLR**	1.64 ± 0.4 a	5.4 ± 3.5 b	1.36 ± 0.58 c	101.677	< 0.001*	0.554
**RBCs count**(million/mcL)	4.92 ± 0.58 a, b	4.86 ± 0.65 a	5.08 ± 0.58 b	4.842	0.025*	0.056
**Hemoglobin (gm/dL)**	13.4 ± 1.7	13.2 ± 1.8	13.2 ± 1.2	1.192	0.286	0.014
**Hematocrit (%)**	39.5 ± 5.2	38.8 ± 5.2	39.8 ± 3.5	2.014	0.157	0.024
**RDW (%)**	12.2 ± 1.3 a	14.2 ± 1.7 b	13.9 ± 1.6 b	58.886	< 0.001*	0.418
**Platelet count** (×10³/ mcL)	235 ± 89 a	295 ± 133 b	257 ± 59 c	21.907	< 0.001*	0.211
**Serum calcium**(mg/dL)	9.88 ± 0.76 a	9.04 ± 0.50 b	9.23 ± 0.62 c	92.910	< 0.001*	0.531
**Serum magnesium**(mg/dL)	2.03 ± 0.17 a	2.02 ± 0.19 a	2.16 ± 0.19 b	33.720	< 0.001*	0.291
**Serum phosphorus**(mg/dL)	3.7 ± 0.57 a	4.5 ± 0.52 b	4.06 ± 0.55 c	107.950	< 0.001*	0.568
**ALT**(IU/L)	29.7 ± 10.2 a	75.4 ± 28.8 b	49.4 ± 19.9 c	225.647	< 0.001*	0.733
**AST**(IU/L)	25.8 ± 9.05 a	66.4 ± 27.1 b	55.2 ± 22.4 c	198.249	< 0.001*	0.707
**GGT**(IU/L)	42.6 ± 13.3 a	87 ± 30 b	69.7 ± 22.7 c	229.646	< 0.001*	0.737
**Serum bilirubin**(mg/dL)	1.09 ± 0.14	0.99 ± 0.22	1.0 ± 0.15	0.556	0.543	0.007
**Serum albumin**(g/dL)	4.2 ± 0.26	4.3 ± 0.38	4.3 ± 0.53	1.334	0.263	0.016
**ALP**(IU/L)	63 ± 21.1 a	97.6 ± 28.5 b	85.9 ± 27.4 c	171.482	< 0.001*	0.677
**BUN** (mg/dL)	15.5 ± 4.1 a	26.9 ± 3.8 b	22.8 ± 4.6 c	343.564	< 0.001*	0.807
**Serum creatinine**(mg/dL)	1.09 ± 0.12 a	1.22 ± 0.18 b	1.07 ± 0.19 a	49.030	< 0.001*	0.374
**eGFR** (ml/ min/1.73 m²)	71.2 ± 14.8 a	63.3 ± 15.4 b	74.2 ± 19.6 a	28.213	< 0.001*	0.256
**Urine ACR** (mg/g)	57.9 ± 43.9 a	108.3 ± 65.9 b	81.4 ± 51.2 c	142.427	< 0.001*	0.635
**VitaminD3** (ng/mL)	21 ± 5 a	24.5 ± 7.5 b	37.3 ± 7.6 c	241.231	< 0.001*	0.746
**Intact PTH** (pg/mL)	54.8 ± 25 a	60 ± 26.8 b	56.5 ± 25.6 a	13.077	< 0.001*	0.138
**HbA1c** (%)	7.02 ± 0.41 a	7.4 ± 0.5 b	8.4 ± 0.47 c	376.968	< 0.001*	0.821
**Total cholesterol** (mg/dL)	215.7 ± 34.8	206.4 ± 37.4	210 ± 31.4	3.303	0.071*	0.039
**Triglycerides**(mg/dL)	266.7 ± 61.7 a	278.2 ± 79.7 b	215.2 ± 58.5 c	27.055	< 0.001*	0.248
**HDL-C**(mg/dL)	42.4 ± 7.1 a	39.4 ± 7 b	43.1 ± 9.2 c	5.698	0.016*	0.065
**LDL-C**(mg/dL)	119.3 ± 31.6 a	110.3 ± 34.4 b	125 ± 29.4 a	8.563	0.004*	0.095
**VLDL** (mg/dL)	51.1 ± 15.4 a	56 ± 17.2 b	42.7 ± 11.2 c	26.057	< 0.001*	0.241
**TG/HDL-C ratio**	6.5 ± 2.2 a	7.3 ± 2.6 b	5.3 ± 2 c	21.376	< 0.001*	0.207
**Serum uric acid**(mg/dL)	6.8 ± 0.87 a	7.8 ± 1.4 b	6.2 ± 1.0 c	93.143	< 0.001*	0.532

¶ Test of significance: One-Way Repeated-Measures ANOVA. † body mass index (BMI), systolic and diastolic blood pressure (SBP&DBP), Mean arterial blood pressure (Mean ABP), erythrocyte sedimentation rate (ESR), C-reactive protein (CRP), white blood cells (WBCs), Absolute neutrophil count (ANC), neutrophil to lymphocyte ratio (NLR), derived neutrophil to lymphocyte ratio (dNLR), red blood cells (RBCs), red blood cell distribution width (RDW), alanine aminotransferase (ALT), aspartate aminotransferase (AST), gamma glutamyl transpeptidase (GGT), alkaline phosphatase (ALP), blood urea nitrogen (BUN), estimated glomerular filtration rate (eGFR), urine albumin/ creatinine ratio (urine ACR), intact parathyroid hormone (Intact PTH), glycosylated hemoglobin (HBA1c), high-density lipoprotein cholesterol (HDL-C), low-density lipoprotein cholesterol (LDL-C), very low-density lipoprotein cholesterol (VLDL-C), triglycerides to high-density lipoprotein cholesterol (TG/HDL-C) ratio. *Pairwise comparisons are represented in letters; a, b &c (similar letters = insignificant difference, while different letters = significant difference); p ≤ 0.05. Bonferroni correction was used for multiple comparisons.

**Table 3:** Categorical repeatedly measured parameters

Parameter	Before	During	After	P value
**Micro-albuminuria** **Pairwise**	59 (71.1%) A	82 (98.8%) B	77 (92.8%) A	< 0.001*
**TG/HDL-C**				0.002*
**<3** **≥3** **Pairwise**	1 (1.2%) 82 (98.8%) A	1 (1.2%) 82 (98.8%) A	9 (10.8%) 74 (89.2%) B	
**RAAS-I use** **Pairwise**	48 (57.8%) A	62 (74.7%) B	71 (85.5%) C	< 0.001*

¶Test of significance: Cochran’s Q test. †Data expression: N (%), †Triglycerides to high-density lipoprotein cholesterol (TG/HDL-C) ratio, †renin angiotensin aldosterone system inhibitors (RAAS- I), *A, B & C to reveal significant variation if different; p ≤ 0.05

**Table 4:** Effect of the interaction between time factor and albuminuria on the studied parameters

Parameter	Normo-albuminuria			Microalbuminuria			Time*Group interaction		
	Before	During	After	Before	During	After	F value	P value	Partial η^2^
**BMI** (kg/m^2^)	28.1 ± 2.7	28.37± 2.75	28.96± 2.80	29.89± 3.60	30.47± 3.67	30.89± 3.50	1.601	0.209	0.019
**SBP** (mmHg)	135.2 ± 9.3	132.29± 9.08	132.50± 9.20	134.75± 7.95	131.69± 8.98	134.66± 9.37	1.052	0.352	0.013
**DBP** (mmHg)	83.5 ± 4.8	82.50± 5.71	85.21± 6.33	85.08± 6.12	80.93± 5.45	84.58± 8.110	1.344	0.264	0.016
**MAP** (mmHg)	100.8 ± 5.8	99.10± 6.35	100.97± 6.36	101.64± 6.24	97.85± 6.0	101.27± 8.08	0.737	0.480	0.009
**Haemoglobin** (g/dL)	13.3 ± 1.9	13.0± 1.82	13.15± 1.35	13.47± 1.69	13.24± 1.74	13.20± 1.09	0.134	0.875	0.002
**HBA1c** %	6.96 ± 0.36	7.35± 0.49	8.34± 0.35	7.0± 0.43	7.42± 0.50	8.45± 0.51	0.042	0.946	0.001
**BUN** (mg/dL)	13.96 ± 3.5	25.21± 2.85	21.04± 3.99	16.19± 4.16	27.64± 3.95	23.51± 4.618	0.035	0.941	0.000
**Serum creatinine**(mg/dL)	1.05 ± 0.11	1.13± 0.15	0.98± 0.13	1.10± 0.11	1.25± 0.17	1.10± 0.19	2.906	0.065	0.035
**eGFR** (ml/ min/1.73 m²)	72.33± 12.31	67.04± 14.09	79.0± 14.13	70.80± 15.80	61.75± 15.69	72.27± 21.21	1.338	0.260	0.016
**Serum uric acid**(mg/dL)	6.84± 0.812	7.82± 1.17	6.40± 1.05	6.76± 0.90	7.72± 1.472	6.14± 0.98	0.308	0.736	0.004
**Total cholesterol**(mg/dL)	223.3 ± 36.9	212.9 ± 41.2	212.7 ± 32.6	212.7 ± 33.7	203.7 ± 35.8	208.9 ± 31.1	0.389	0.545	0.005
**Triglycerides**(mg/dL)	260.5± 57.77	270.0± 59.55	216.75± 66.7	269.24± 63.5	281.46± 86.8	214.61± 55.4	0.252	0.653	0.003
**HDL-C** (mg/dL)	42.67± 6.91	38.0± 6.21	42.92± 12.09	42.29± 7.23	39.98± 7.23	43.22± 7.81	0.442	0.530	0.005
**LDL-C**(mg/dL)	127.17± 31.9	119.25± 35.3	125.50± 27	116.14± 31.2	106.61± 33.7	124.81± 30.5	1.345	0.252	0.016
**VLDL** (mg/dL)	49.92± 15.83	55.17± 14.84	43.17± 12.20	51.63± 15.33	56.27± 18.21	42.44± 10.91	0.188	0.698	0.002
**TG / HDL**	6.20± 1.50	7.23± 1.755	5.52± 2.38	6.67± 2.44	7.32± 2.82	5.19± 1.80	0.667	0.433	0.008
**NLR**	2.35± 0.67	11.83± 7.46	1.99± 0.93	2.10± 0.68	9.60± 7.97	1.78± 0.80	1.111	0.296	0.014
**dNLR**	1.71± 0.38	5.93± 3.23	1.45± 0.62	1.6± 0.43	5.18± 3.61	1.32± 0.55	0.554	0.462	0.007
**WBCs** (× 10^9^/L)	6.56± 1.42	7.55± 2.57	6.19± 1.63	6.27± 1.72	7.57± 3.62	6.62± 1.95	0.703	0.467	0.009
**ANC**(×10^3^/mcL)	4.10± 1.03	6.31± 2.50	3.49± 1.0	3.80± 1.16	6.07± 3.55	3.65± 1.33	0.328	0.618	0.004
**Albumin**(g/dL)	4.25± 0.208	4.37± 0.347	4.41± 0.481	4.20± 0.27	4.24± 0.39	4.20± 0.53	1.428	0.243	0.017
**ALP**(IU/L)	67.79± 19.77	111.71± 28.3	96.71± 25.28	61.07± 21.53	91.81± 26.74	81.56± 27.19	3.131	0.062	0.037
**Vitamin D3** (ng/mL)	19.29± 4.99	24.0± 5.34	36.58± 9.141	21.69± 4.89	24.64± 8.23	37.64± 7.014	0.562	0.553	0.007
**Intact PTH** (pg/mL)	39.39± 20.96	42.19± 19.23	40.17± 18.86	60.99± 23.92	67.33± 26.18	63.11± 25.05	1.165	0.309	0.014
**Calcium**(mg/dL)	10.16± 0.53	9.07± 0.41241	9.33± 0.49	9.76± 0.80	9.02± 0.53	9.18± 0.66	2.198	0.121	0.026
**Magnesium**(mg/dL)	2.01± 0.14	2.00± 0.19	2.12± 0.20	2.03± 0.183	2.03± 0.18	2.16± 0.19	0.164	0.826	0.002
**Phosphorus**(mg/dL)	3.69± 0.62	4.55± 0.39	4.10± 0.36	3.75± 0.54	4.49± 0.56	4.04± 0.60	0.687	0.481	0.008

¶Test: two-way mixed ANOVA, † body mass index (BMI), systolic and diastolic blood pressure (SBP&DBP), Mean arterial blood pressure (Mean ABP), glycosylated hemoglobin (HBA1c), blood urea nitrogen (BUN), estimated glomerular filtration rate (eGFR), high-density lipoprotein cholesterol (HDL-C), low-density lipoprotein cholesterol (LDL-C), very low-density lipoprotein cholesterol (VLDL-C), triglycerides to high-density lipoprotein cholesterol (TG/HDL-C) ratio, neutrophil to lymphocyte ratio (NLR), derived neutrophil to lymphocyte ratio (dNLR), white blood cells (WBCs), Absolute neutrophil count (ANC), alkaline phosphatase (ALP), intact parathyroid hormone (Intact PTH), * significant value; p ≤ 0.05. Bonferroni correction was used for multiple comparisons

**Table 5:** Main effects of albuminuria and time factor on the studied parameters

Parameter	Main effect of albuminuria			Main effect of time		
	F value	P value	Partial η^2^	F value	P value	Partial η^2^
**BMI** (kg/m^2^)	5.682	0.019*	0.066	49.07	< 0.001*	0.377
**SBP** (mmHg)	0.043	0.836	0.001	3.880	0.023*	0.046
**DBP** (mmHg)	0.043	0.867	0.001	6.039	0.003*	0.069
**MAP** (mmHg)	0.000	0.985	0.000	5.911	0.003*	0.068
**Hemoglobin** (g/dL)	0.263	0.609	0.003	0.902	0.408	0.011
**HBA1c** %	0.869	0.354	0.011	303.7	< 0.001*	0.789
**BUN** (mg/dL)	8.755	0.004*	0.098	276.8	< 0.001*	0.774
**Serum creatinine**(mg/dL)	8.214	0.005*	0.092	39.11	< 0.001*	0.326
**eGFR** (ml/ min/1.73 m²)	1.640	0.204	0.020	23.66	< 0.001*	0.226
**Serum uric acid**(mg/dL)	0.408	0.525	0.005	73.06	< 0.001*	0.474
**Total cholesterol**(mg/dL)	1.279	0.261	0.016	3.036	0.083	0.036
**Triglycerides**(mg/dL)	0.272	0.603	0.003	20.10	< 0.001*	0.199
**HDL-C**(mg/dL)	0.293	0.590	0.004	5.809	0.015*	0.067
**LDL-C**(mg/dL)	1.725	0.193	0.021	5.066	0.025*	0.059
**VLDL** (mg/dL)	0.066	0.798	0.001	19.86	< 0.001*	0.197
**TG / HDL**	0.040	0.842	0.000	15.76	< 0.001*	0.163
**NLR**	1.897	0.172	0.023	83.60	< 0.001*	0.508
**dNLR**	1.230	0.271	0.015	88.94	< 0.001*	0.523
**WBCs** (× 10^9^/L)	0.013	0.910	0.000	9.630	< 0.001*	0.106
**ANC**(×10^3^/mcL)	0.112	0.739	0.001	43.27	< 0.001*	0.348
**Albumin**(g/dL)	2.657	0.107	0.032	2.046	0.145	0.025
**Vitamin D3**(ng/mL)	1.084	0.301	0.013	201.6	< 0.001*	0.713
**Intact PTH** (pg/mL)	17.39	< 0.001*	0.177	7.998	< 0.001*	0.090
**ALP**(IU/L)	8.645	0.004*	0.096	170.18	< 0.001*	0.678
**Calcium**(mg/dL)	4.494	0.037*	0.053	91.97	< 0.001*	0.532
**Magnisium**(mg/dL)	0.676	0.413	0.008	25.70	< 0.001*	0.241
**Phosphorus**(mg/dL)	0.027	0.870	0.000	93.90	< 0.001*	0.537

¶Test: two-way mixed ANOVA, † body mass index (BMI), systolic and diastolic blood pressure (SBP&DBP), Mean arterial blood pressure (Mean ABP), estimated glomerular filtration rate (eGFR), glycosylated hemoglobin (HBA1c), white blood cells (WBCs), neutrophil to lymphocyte ratio (NLR), derived neutrophil to lymphocyte ratio (dNLR), † ANC = Absolute neutrophil count, blood urea nitrogen (BUN), intact parathyroid hormone (intact PTH), alkaline phosphatase (ALP), Triglycerides (TG), very low-density lipoprotein cholesterol (VLDL-C), and triglycerides to high-density lipoprotein cholesterol (TG/HDL-C) ratio, low-density lipoprotein cholesterol (LDL-C), * significant value; p ≤ 0.05

## Discussion

In the current study, Albuminuria was prevalent in 71% of patients before COVID-19 and subsequently prevailed in 92.8% after recovery. The highest level of albuminuria prevalence was noticed during COVID-19 with partial subsiding without achieving pre-COVID level. Interestingly, urine ACR remained within the micro-albuminuria range throughout the study. Kidneys are tackled during COVID-19 due to the direct invasion of SARS-CoV2 via ACE2 receptors located in the epithelium of the proximal renal tubules [18]. Our results agreed with Antoine et al., [19] who reported persistent mild albuminuria with slightly improved eGFR for 50 days following recovery of COVID-19. Xu-Wei et al. [20] reported a partial resolution of serum creatinine, BUN, and albuminuria within 30 days after resolution of COVID-19. Meanwhile, Pei et al. [21] noticed complete recovery of proteinuria in the majority of COVID-19 survivors within 3 weeks due to resolution of transient proteinuria induced by fever and sepsis rather than kidney injury. Inconsistent results could be explained by different study design and methods of albuminuria assessment. Xiaoying et al. [22] noticed worsened kidney functions within the consequent year after recovery in patients who confronted COVID-19 with acute kidney injury (AKI). Fortunately, none of our patients developed acute kidney injury throughout the study even with persistent and progressive increased prevalence of microalbuminuria. Our results are consistent with Ki Ryange et al. [23] who noticed reduced prevalence of AKI in patients with COVID-19 despite concomitant increased albuminuria. Baseline albuminuria independently determined severity and poor outcomes of COVID-19. [24–27] In alignment with the current study, patients with albuminuria exhibited more frequent severe COVID-19 with more frequent hospitalization. Disrupted glomerular membrane integrity, proximal tubular brush border injury, micro-thrombosis, necrosis, and vacuolar degeneration were postulated to explain COVID-19 associated proteinuria [28]. Despite persistent and increased prevalence of microalbuminuria for 1 year after recovery in more than 92% of our participants, we noticed complete resolution of serum creatinine and eGFR. Sub-clinical kidney injury and absence of AKI among our participants could explain complete recovery of glomerular filtration.

In overall participants, we noticed progressively increased HBA1c level during and after recovery than before COVID-19. In agreement with Rimesh et al. [29] and Müller J.A et al. [30] who attributed worsened glycemic control during and after COVID-19 recovery to direct and indirect adverse effects of SARS-CoV2. Direct invasion and replication of SARS-CoV2 in the pancreatic cells may induce detrimental long-term effects on the beta-cell alongside impaired insulin secretion. Moreover, administration of systemic steroids to improve the outcomes and accelerate recovery may enhance steroid-induced hyperglycemia. In consistency with Liu X. et al. [31] who reported steroid-induced hyperglycemia in more than one-third of steroid-treated patients. Montefusco et al. [32] reported disrupted plasma glucose homeostasis among COVID-19 survivors 2 months after recovery that was explained by COVID-19-related cytokine storm, insulin resistance, and beta-cell dysfunction. Furthermore, Shimona Starling [33] noticed persistent hyperglycemia for 6 months after COVID-19 recovery in approximately 35% of patients who developed new-onset hyperglycemia during COVID-19.

In the current study, dyslipidemia was evident in 88% of participants. Alterations of lipid profiles were relevant throughout the study. Triglycerides (TG), very low-density lipoprotein-cholesterol (VLDL-C), and triglycerides to high-density lipoprotein-cholesterol (TG/HDL-C) ratio were significantly elevated during COVID-19 that progressively improved after recovery. On the contrary, low-density lipoprotein-cholesterol (LDL-C) was initially lowered during COVID-19 but significantly elevated after recovery. Our results agreed with Li Y, et al. [34] who reported lowered total cholesterol and LDL-C among patients with COVID-19. They noticed a gradual rise of LDL-C levels with resolution of COVID-19. Furthermore, Roshan et al. [35] and Chen et al. [36] reported an inverse correlation between total cholesterol, LDL-C, HDL-C with the duration of hospital stay, the severity and adverse outcomes of COVID-19. Sequential rises of LDL-C and HDL-C during COVID-19 were considered as promising markers of recovery. Furthermore, Mohammed et al. [37] considered concomitant lowered total cholesterol (TC), LDL-C, and HDL-C alongside elevated TG as predictors of exaggerated cardiovascular risks in patients with COVID-19. Our results disagreed with Roccaforte et al. [38] who reported progressively increased levels of TG after recovery meanwhile our participants showed a gradual decline in TG/HDL and VLDL-C levels 1 year after recovery. Variations in study design, population, and methodology could explain this inconsistency. SARS-CoV2 related systemic inflammatory response, macrophage activation, and pro-inflammatory cytokines surge may explain COVID-19 associated altered serum lipid profile. Lipids are fundamental for viral entrance, endocytosis, replication, fuel production, and intracellular signaling [39]. In our study, NAFLD was prevalent in 12% of participants. Non-significant alterations of serum bilirubin and serum albumin were found among participants before, during, and after COVID-19. Nonetheless, serum ALT, AST, GGT, and ALP were statistically and significantly elevated during COVID-19 than before contracting infection or after recovery. We noticed a gradual improvement in ALT, AST, GGT, and ALP after recovery but did not achieve pre-COVID-19 levels. Marjot, et al. [40] noticed liver injury among 65% of patients with COVID-19. Furthermore, Gan et al. [41] reported persistently deranged liver functions in approximately 40% of patients at the time of discharge after COVID-19 recovery. **Xuejiao et al.** [42] reported sequential improved prevalence of deranged liver functions from 35.3%, 25.7%, 15.6%, 15%, to 10.8% at discharge time, after 1, 3, 6, and 12-months after recovery, respectively. In China, **Liu, et al.** [43] noticed disrupted liver functions in more than 7% of patients within the first year after COVID-19 recovery. **Ya-Wen et al.** [44] reported normalization of ALT, GGT, and ALP within the consecutive 60 days of COVID-19 recovery. In the current study, the main effect of albuminuria regardless of time factor and the main effect of time factor regardless of albuminuria were relevant on ALP. Therefore, ALP is one of the parameters that should be looked for with frequent assessment after COVID-19 recovery, in particular in patient with T2D and albuminuria. Obviously, our participants did not show significant alterations in serum bilirubin throughout the study. In agreement with **Fan et al.** [45] who denied elevation of serum bilirubin in patients with COVID-19. COVID-19 associated systemic inflammatory response, cytokines surge, pneumonia-related hypotension and hypoxemia, drug-induced hepatic injury, micro-vesicular steatosis, portal inflammatory cell-infiltrate, lymphocytic endothelitis, and direct SARS-CoV2 cellular invasion may explain COVID-19-related liver injury [46, 47].

Interestingly, levels of vitamin D3 were statistically and significantly improved after recovery. Improved vitamin D3 level may be explained by the strict adherence to vitamin supplementation with a healthy lifestyle during and after recovery. Serum calcium was significantly lowered during acute COVID-19 than before COVID-19 or after recovery. Wessam et al. [48] reported lowered serum calcium in 67% of diabetic patients with COVID-19 and in 78% of COVID-19 patients admitted to ICU that represented an independent predictor of poor outcomes. Despite lack of the effect of the interaction between time factor and albuminuria, serum calcium was significantly influenced by time factor regardless of albuminuria and by albuminuria factor regardless of time point. Therefore, serum calcium should be included in the long-term follow-up medical care of COVID-19 survivors with T2D and albuminuria. Disrupted serum calcium and urine albumin excretion are considered as modifiable risk factors for CKD progression that may help in early preventive measures [49] serum calcium is tightly linked to altered vitamin D3 and intact PTH levels in patients with DM and diabetic nephropathy. Progressive albuminuria may adversely affect serum calcium level through disordered serum albumin, Vitamin D3 synthesis, metabolism, and action [50, 51]. Therefore, it was plausible to search for altered serum calcium and albuminuria in patients with T2D with COVID-19 experience. Serum calcium is crucial in viral replication. Exaggerated combining of excess-free and unsaturated fatty acids with the ionized calcium during COVID-19 may induce hypocalcemia. Furthermore, lowered calcium level may precipitate mitochondrial dysfunctions and cytokine storm that drive poor outcomes of COVID-19 [52]. In the current study, we noticed significant alterations of systemic inflammatory markers such as ESR, CRP, and ferritin being highest during COVID-19 with partial improvement after recovery. Therefore, a residual low-grade inflammation was evident in patients with T2D for one year after COVID-19 recovery. In consistency with other researchers who noticed elevated CRP, D-dimer, and ferritin for one to three months after recovery.[53] On the other side, although there were significant alterations in the lymphocytic count, NLR, and derived NLR throughout the study timepoints, we noticed complete resolution of the lymphocytic count, NLR, and derived NLR after recovery.

Our participants exhibited reduced lymphocytic count with elevated NLR, and derived NLR during COVID-19 illness. Lowered lymphocytic count and elevated NLR significantly predicted poor outcomes and severity of COVID-19 [54, 55]. Generous expression of ACE2 receptors on the lymphocytes may mediate direct invasion and damage of lymphocytes by SARS-CoV2. Moreover, the exacerbated systemic inflammatory response and cytokine storm can exacerbate apoptosis and atrophy of the lymphoid system [56]. Obviously, the mean arterial blood pressure was significantly elevated in our participants after recovery than before or during COVID-19. Arterial hypertension was more prevalent among patients with albuminuria than patients without albuminuria (66.1% versus 37.5%). Moreover, the proportion of RAAS-I users increased robustly from 57.8% before COVID-19 to 85.5% after recovery. The main effect of time factor was significantly obvious on mean ABP. Exaggerated fear, anxiety of the pulmonary residues, symptoms of post-COVID-19 syndrome, socio-economic burdens, and physical impairment may explain alteration of arterial blood pressure. Our results agreed with Rachel et al. [57] who noticed a significant elevation of a systemic arterial stuffiness after recovery. In our participants, Bodyweight and BMI were significantly increased after recovery. Moreover, the main effects of time factor and albuminuria were relevant on BMI throughout the study. Therefore, altered BMI should be addressed in COVID-19 survivors with T2D and albuminuria. Abstinence of physical activity due to post-COVID-19 depression, fatigue, and mild exertion related-dyspnea may explain the progressive rise of BMI throughout the 3 time points of the study. In addition, administration of medicine with weight-gain adverse effects like steroids and the intensified therapy for strict control of hyperglycemia could explain progressively increased bodyweight and BMI. On the contrary, Didier et al. [58] noticed weight loss in COVID-19 survivors particularly in patients with severe COVID-19 and renal impairment. Weight loss in their cohort was attributed to appetite suppression and impaired taste sensation which may persist after recovery. In the current study, we noticed lack of simple main effects for the interaction between time factor and albuminuria on the studied parameters, nevertheless, the main effects of time factor regardless of albuminuria and the main effects of albuminuria regardless of time point were relevant for multiple parameters. Therefore, the long-term follow-up protocol of care of COVID-19 survivors with T2D should include BMI, mean ABP, HBA1c, BUN, serum creatinine, eGFR, serum albumin, lipid profile, intact PTH, vitamin D3, ALP, serum calcium.

The current study confronted multiple limitations like the single-ethnicity, single-center design, dropped-out cases for missing data but with preserved sample size validity. However, to our knowledge, little is announced about the potential effects of time and albuminuria on morbid alterations among patients with T2D before, throughout, and 1 year after COVID-19 recovery. We strived to avoid confounders of albuminuria like concomitant fever, exercise, sepsis, menstruation, and drug-induced acute tubular necrosis before and after COVID-19. Nonetheless, during COVID-19, albuminuria of fever and SARS-CoV2-related sepsis could not be differentiated from albuminuria of diabetic nephropathy. Finally, the invasive tools to detect molecular and cellular changes during and after COVID-19 recovery were not available. Future larger studies with multi-center design and multiple ethnicities are warranted to enforce our results. We had multiple strength points like the longitudinal comprehensive design to track and address alterations of various clinical and biochemical characteristics before, during, and 1 year after recovery. Literature review have a lot of studies dealt with SARS-CoV2-induced changes during and after recovery without backward interest. Moreover, studying the result of the influence of time factor (3 time points), albuminuria, and their interaction on the studied parameters revealed relatively new data that can beneficial in our clinical approach. Our results may be considered in the long-term medical care protocols of COVID-19 survivors with T2D.

## Conclusion

The clinical and biochemical characteristics of patients with T2D and COVID-19 showed significant alterations throughout the study. Time factor and albuminuria exerted relevant main effects on the patients’ characteristics that should be considered in the medical care practice.

## Data Availability

The data set generated and/or analyzed during this research are available from the corresponding author upon a reasonable request.

## Abbreviations

T2D: Type 2 diabetes mellitus
SARS-CoV-2: Severe acute respiratory syndrome coronavirus 2
ACE2: Angiotensin-converting enzyme 2
DM: Diabetes mellitus
RT-PCR: Real time polymerase chain reaction
BUN: Blood urea nitrogen
eGFR: Estimated glomerular filtration rate
urine ACR: Urine albumin/ creatinine ratio
ALT: Alanine aminotransferase
AST: Aspartate aminotransferase
GGT: Gamma glutamyl transpeptidase
ALP: Alkaline phosphatase
HBA1c: Glycosylated hemoglobin
ESR: Erythrocyte sedimentation rate
CRP: C-reactive protein
NLR: Neutrophil to lymphocyte ratio
dNLR: Derived neutrophil to lymphocyte ratio
NAFLD: Non-alcoholic fatty liver disease
ICU: Intensive care unit
RAAS- I: Renin angiotensin aldosterone system inhibitors
i PTH: Intact parathyroid hormone
TG: Triglycerides
VLDL-C: Very low-density lipoprotein cholesterol
TG/HDL-C: Triglycerides to high-density lipoprotein cholesterol ratio
LDL-C: Low-density lipoprotein cholesterol
BMI: Body mass index
ADRS: Acute respiratory distress syndrome
